# Prosthetic Rehabilitation of Child Victim of Avulsion of Anterior Teeth with Orthodontic Mini-Implant

**DOI:** 10.1155/2017/8905965

**Published:** 2017-09-28

**Authors:** Natalice Sousa de Oliveira, Gabriella Lopes de Rezende Barbosa, Lincoln Dias Lanza, Henrique Pretti

**Affiliations:** ^1^Department of Pediatric Dentistry and Orthodontics, Division of Orthodontics, School of Dentistry, Federal University of Minas Gerais, Belo Horizonte, MG, Brazil; ^2^Department of Oral Diagnosis, Division of Oral Radiology, Piracicaba Dental School, University of Campinas, Piracicaba, SP, Brazil; ^3^Department of Restorative Dentistry, School of Dentistry, Federal University of Minas Gerais, Belo Horizonte, MG, Brazil; ^4^Department of Pediatric Dentistry and Orthodontics, Division of Pediatric Dentistry, School of Dentistry, Federal University of Minas Gerais, Belo Horizonte, MG, Brazil

## Abstract

The treatment of choice in cases of avulsed permanent teeth is the immediate reimplantation. However, this conduct does not always work favorably, either by failures in the initial approach or by inappropriate interventions. In this sense, the aim of this study is to present an alternative prosthetic rehabilitation with the use of orthodontic mini-implants in the anterior region. This case reports a ten-year-old child with history of avulsion of superior central incisors. The therapeutic approach was planned to promote physiological teeth contacts and acceptable esthetics and phonetics. First, the occlusal-gingival insertion of two orthodontic mini-implants was performed in the alveolar ridge, and, immediately after that, two provisional crowns were attached to the implants. The interventions achieved satisfactory cosmetic and functional results. After one-year follow-up, the adjacent periodontal tissues remained without signs and/or symptoms of inflammation. The provisional crowns presented no mobility and fractures. During radiographic examination, a healthy bone tissue appearance was observed. The simplicity of mini-implant installation makes them a promising alternative for temporary prosthetic rehabilitation of patients undergoing growth and development. The technique provides positive aesthetic and functional results that may reflect on self-esteem and social inclusion of children and adolescents.

## 1. Introduction

Traumatic accidents that result in avulsion of anterior teeth can have a negative impact on the quality of life of the child and family [1]. When replantation fails, potential sequelae have been reported, usually associated with aesthetics, quality of life, nutrition, phonetics, arch integrity, and oral habits development [2]. In these circumstances, the multidisciplinary therapeutic approach handles the best periodontal, occlusal, aesthetic, and functional results [3].

At an early age, the use of orthodontic mini-implants to support crowns proves to be a promising alternative, due to contraindication for rehabilitation with osseointegrated dental implants and disadvantages of removable dentures. It is an inexpensive technology resource and minimally invasive and easy clinical applicability. This case report presents an immediate prosthetic rehabilitation with the use of orthodontic mini-implants in a patient with avulsion of anterior teeth.

## 2. Case Report

**Figure 1:**
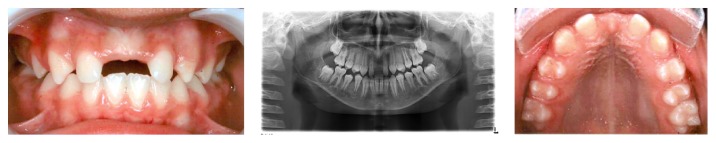
Clinical and radiographic aspects of the edentulous region with transverse deficiency due to mesialization of the lateral incisors.

Ten-year-old male patient, with absence of maxillary central incisors, presented to the Orthodontics Department at Federal University of Minas Gerais, complaining of aesthetic dissatisfaction associated with social awkwardness. The patient reported history of dental avulsion of the anterior teeth which had occurred three years earlier without reimplantation attempt. Clinical examination revealed edentulous region with intact mucosa, no apparent bony defect, and satisfactory vertical dimension of occlusion and maxillomandibular sagittal relationship. However, the patient presented with transverse deficiency of the premaxilla (Figure 1). The treatment goal was to promote physiological teeth contacts and acceptable esthetics and phonetics. The treatment plan consisted in installation of two orthodontic mini-implants in the remaining alveolar ridge for immediate support of two provisional crowns.

The executed treatment consisted of the following steps.

**Figure 2:**
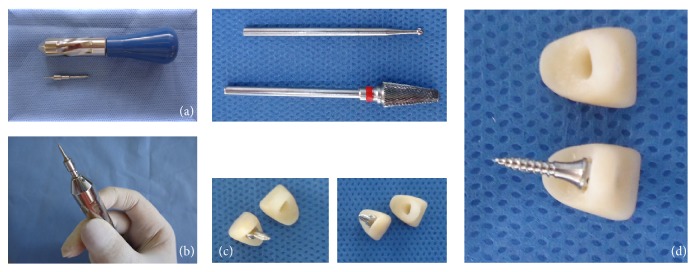
Manual instrument (Morelli, Sorocaba, São Paulo, Brazil) ((a) and (b)); anatomical conformation of temporary crowns (c); coupling test of the mini-implant and cavity created in the crown (d).

**Figure 3:**
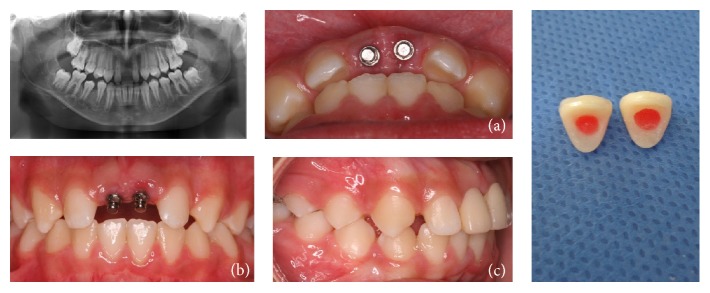
Edentulous region after mini-implants insertion ((a) and (b)); temporary crowns adapted to the heads of the mini-implants (c).

1. Selection of mini-implants (Figure 2): two mini-implants, both of similar geometry, self-tapping and self-drilling, made of titanium alloy with dimensions of 1.6 mm diameter and 10 mm length (Morelli, Sorocaba, São Paulo, Brazil) were selected. Dental implant kit with manual instrument was also provided.
2. Preparation of temporary crowns (Figure 2): two false teeth received morphological adaptation using acrylic resin, in order to resemble the anatomy of the lost elements and adapt to the remaining gum line conformation. In the central part of the framed region, cavities of equivalent dimensions of the mini-implant head were created. A model with similar geometry of the chosen mini-implants was used for this test.
3. Adequacy of the oral environment: prophylaxis of oral cavity with chlorhexidine gluconate 0.12% and local anesthesia of the edentulous region (topical-lidocaine 5% and submucous-lidocaine 2%).
4. Installation of mini-implants (Figure 3): the insertion of the implants was transmucosal in occlusal-gingival position. The adaptation adjustments to the alveolar ridge and the three-dimensional orientation of the crowns were conducted with acrylic resin with intermittent removal/insertion movements, in order to prevent undesirable retention of the resin.
5. Final retention of provisional crowns to mini-implants (Figure 3): The final coupling was completed with the partial filling of the cavity (approximately 1 mm below the alveolar ridge) with acrylic resin, still in plastic phase, to allow mechanical interlock between the crowns and heads of mini-implants after setting time. It was important to ensure that the transmucosal profile region remained completely free of any excess of resin. This procedure was performed using dental floss moistened with liquid monomer for cleaning potential spillovers.

After this step, occlusal adjustment was executed in order to promote physiological contacts in maximum intercuspation and eccentric movements. Patient and family were informed about the hygiene of the oral cavity with emphasis on flossing the peri-implant region. They were also advised about restrictions in food apprehension and cut, especially bread, meat, and fruit, which should be divided into small pieces to minimize axial forces action on the mini-implants.

## 3. Results

**Figure 4:**
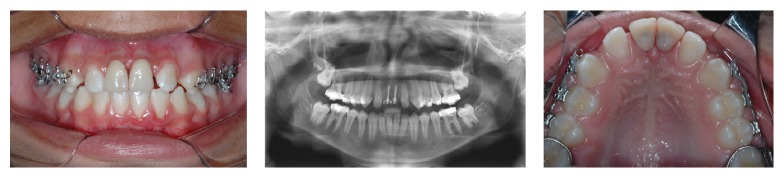
Clinical and radiographic aspects 6 months after mini-implant placement.

**Figure 5:**
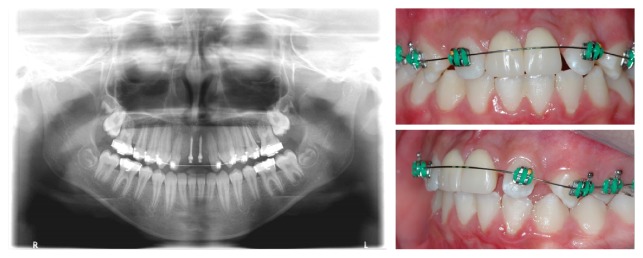
Clinico- and radiographic aspects one year after mini-implant placement.

Satisfactory aesthetic and functional results were achieved. Monthly clinical follow-up and radiographic examination every three months after installation of mini-implants were done. In the follow-up period of one year, the adjacent periodontium presented without inflammatory signs or symptoms. Moreover, the crowns did not present mobility and a healthy alveolar bone appearance was observed during radiographic evaluation. After the initial prosthetic rehabilitation, the patient was referred to orthodontic treatment with fixed appliances to recover the space loss due to mesial migration of the teeth adjacent to the edentulous region (Figures 4 and 5). Both patient and family members expressed satisfaction with the treatment provided.

## 4. Discussion

The prevalence of anterior dental trauma in the age group 6–17 years old ranges between 4% and 37.9%. Among the resulting injuries, avulsion of permanent teeth occurs in approximately 16% of the cases, and the most affected teeth are the superior incisors [4, 5]. In these circumstances, tooth replantation is usually recommended because it restores aesthetic function, delays the need for prosthetic work, and reduces the psychological impact of immediate loss. However, this conduct does not always work favorably, by either failures in the initial approach or inappropriate interventions [6].

Besides reimplantation, different strategies for anterior prosthetic rehabilitation in children victim of dental avulsion have been described. The most common techniques include attachment of artificial teeth in braces [7], tooth replantation [8], removable partial dentures [9, 10], space closure with fixed orthodontic appliances, followed by reanatomization of lateral incisors and canines [11], and adhesive prosthesis [12].

Little has been addressed regarding the use of mini-implants to support crowns, especially in patients at skeletal maturation period [13]. Unlike conventional dental implants that behave as ankylosed teeth interfering with bone growth [14], orthodontic mini-implants without surface treatment present minimum osseointegration. These implants rarely cause tissue damage when removed [15], a factor that may potentially minimize dimensional losses of alveolar ridges for planning definitive rehabilitation. This low cost technique demands minimally invasive technique and easy clinical applicability; it also gives additional advantages to enable prosthetic rehabilitation intervention in only one appointment.

Nevertheless, the possibility of immediate loading has limitations. Morphological and anatomical characteristics of the insertion site and geometric characteristics of the mini-implant directly influence its stability [16]. Despite the restrictions, especially in relation to food size in the case presented, the direction of insertion (occlusal-gingival in premaxillary alveolar process) and the length of the mini-implants appear to have contributed to treatment success. Additionally, occlusal adjustments that minimize unnecessary occlusal loads, the smooth surface of transmucosal profile that avoids plaque buildup and enables the adequate cleaning of the peri-implant region by flossing, added up to the satisfactory results achieved.

## 5. Conclusion

The versatility and simplicity of mini-implant installation make them a promising alternative to crown anchorage in the anterior region, especially in oral rehabilitation of patients under development. Despite limitations, it is a simple technological resource that allows a low cost intervention in a single appointment. It also provides aesthetic and functional results that can improve the patients' quality of life, reflecting on self-esteem and social integration.
